# COVID-19 and informal settlements – implications for water, sanitation and health in India and Indonesia

**DOI:** 10.14324/111.444/ucloe.000011

**Published:** 2020-09-07

**Authors:** Priti Parikh, Yasmin Bou Karim, Jacob Paulose, Pam Factor-Litvak, Emily Nix, Dewi Nur Aisyah, Hemant Chaturvedi, Logan Manikam, Monica Lakhanpaul

**Affiliations:** 1 University College London, Engineering for International Development Centre, Civil, Environmental and Geomatic Engineering, 2 Taviton Street, London WC1H 0BT, UK; 2 Aceso Global Health Consultants Ltd, London, UK; 3 University College London, Institute for Environmental Design and Engineering, London, UK; 4 Department of Epidemiology, Columbia University, New York, NY, USA; 5 Indonesia One Health University Network, Jakarta, Indonesia; 6 UCL Institute of Epidemiology & Healthcare, London, UK; 7 Population, Policy and Practice, University College London, Great Ormond Street Institute of Child Health, London, UK

**Keywords:** COVID-19, WASH, informal settlements, India, Indonesia, infection pathways, water, the environment, policy and law

## Abstract

Informal settlements are home to over 1 billion people worldwide and are characterised by high population densities and poor environmental conditions. The authors identify the impact of COVID-19 on existing water and sanitation practices and potential pathways for the transmission of COVID-19 in informal settlements in India and Indonesia. In the short term, there is an urgent need for mobile and contactless hand washing, washing/bathing facilities and toilets. In the long term, COVID-19 provides an opportunity to invest in centralised water and sanitation networked solutions appropriate for high-density settings to integrate those settlements into cities and improve environmental conditions and health in these cities.

The influenza pandemic of 1918 (Spanish flu) is considered to be the most lethal pandemic of the 20th century. It resulted in the largest number of deaths in individual countries; high mortality rates were seen in Indonesia and c. 10–20 million fatalities were reported in India [1]. At the time, vaccines, in combination with social measures at the individual, household and societal levels, proved to be effective public health interventions [1]. Now, decades later, COVID-19 poses a similar challenge globally, but the highly contagious nature of the disease and there being no currently available vaccine requires stringent physical distancing measures, involving particular challenges in overcrowded areas.

Ongoing urbanisation and industrialisation processes have contributed to a rapid increase in the number of informal settlements worldwide. These settings are characterised by high population densities and poor environmental conditions and are currently home to over 1 billion people [2]. Residents of overcrowded spaces are also at a higher risk of outbreaks of infections and disease [3]. In light of the COVID-19 pandemic, contextualisation of the interventions required to contain the incidence of infections is important. In areas where overcrowding is the norm and access to outdoor space is limited, adherence to key public health messaging on physical distancing and handwashing is challenging, as individual sanitation facilities are virtually non-existent and access to clean water and soap is limited. For example, in India at least 542 million and in Indonesia 94 million people do not have basic handwashing facilities with soap and water at home [4]. Measures implemented in mainstream cities are therefore difficult to apply to unplanned high-density informal settlements [5].

The authors are members of the Childhood Infections and Pollution (CHIP) Consortium that is aimed at reducing infection and antimicrobial resistance among children under 5 years old who are living in slums. In 2019, the Consortium used tools such as transect walks and focus group discussions to understand pre-pandemic Water Sanitation and Hygiene (WASH) challenges in two settlements in India and Indonesia. Building on this and through consultations with in-country Consortium members during the pandemic, this paper identifies the impact of COVID-19 on existing WASH practices and potential pathways for the transmission of COVID-19 in these slum areas (see Fig. 1). In addition to building on the knowledge and expertise of in-country Consortium members, the in-country co-authors collated qualitative responses to questions on WASH practices pre COVID-19 and during the lockdown in India and large-scale limitations on mobility in Indonesia. The responses were synthesised by the authors and are presented in this paper.

**Figure 1:**
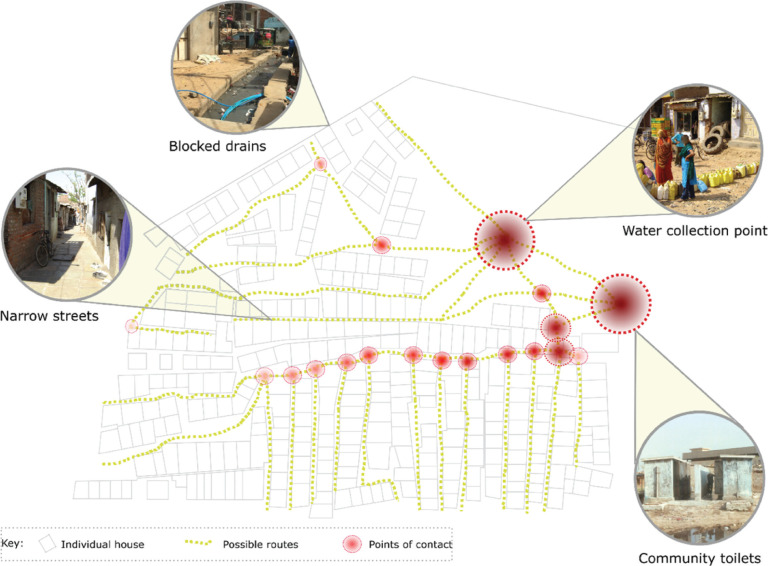
Illustrative representation of potential COVID-19 transmission pathways in slums in India and Indonesia.

During the COVID-19 outbreak, India has been under a lockdown where people’s movements have been restricted to essential outings, and areas with a high incidence of COVID-19 cases have been sealed off by the government. Settlements such as Dharavi, the largest slum in Asia, are now hotspots for COVID-19. In informal settlements, women are typically responsible for water collection and waste disposal, which requires them to frequently leave their homes, as very few dwellings have water connections. These communal points for water collection create additional high-risk transmission pathways, given personal protective equipment (PPE) is not available. In our observational work, we did not see evidence of the water being stored in clean containers, which poses a risk for household transmission of COVID-19 to the elderly and vulnerable in households where multiple generations live together. In addition, leakages from pipes normally result in contamination of water sources; water buckets are not cleaned regularly and standing in long queues for water collection in narrow lanes is not usually accompanied by physical distancing, all of which could lead to further transmission risks.

The majority of residents also practise defecation in open fields, rivers and water streams within or nearby their communities, with limited scope for physical distancing and appropriate hand washing/hygiene measures. Open drains in these settlements are often blocked with solid waste and effluents. As the workers who are typically responsible for maintaining the drains are reluctant to come into the communities during the lockdown, the condition of these channels is deteriorating and higher levels of risk to other infectious diseases are on the horizon. Early evidence suggests that traces of COVID-19 virus have been found in the faeces of some patients diagnosed with COVID-19 as well as in untreated wastewater [6]. It is premature to tell the impact of exposure to human faeces and wastewater; however, it could result in an added risk for COVID-19 infections.

The first line of defence during the current pandemic is handwashing with soap, but in most of the homes visited in our work, soap was not available or, if available, was not used frequently to wash hands. Adding to the complexity of potential infection transmission pathways during the pandemic is the quality of the water used for handwashing. At times, the definition of ‘safe water’ falls short and does not take into account crucial factors such as the quality of the water and risk of contamination, for example, in distribution pipes. Studies have shown that in the past coverage of safe water in India was grossly overestimated when water safety parameters were considered. This means that during the pandemic, the increased promotion and adherence to recommended handwashing practices in communities where the water supplied is at high risk of microbiological and/or other types of contamination might not have the desired public health effect [7,8].

Many of the households in our study were also reliant on public toilets. Women who use public toilets take the risk of enhanced exposure to COVID-19 due to the toilets and associated surfaces being dirty and the close physically proximity to other users. Women using public toilets are often subject to violence which influences how frequently they use those facilities [9]. This can significantly impact their health, with those risks further exacerbated during the pandemic.

In contrast to India, Indonesia has taken a more pragmatic approach to COVID-19 by practising large-scale limitations of mobility instead of a full lockdown. In Indonesian slums, women normally purchase water from travelling vendors who carry water containers in wheelbarrows. During the pandemic, this activity is continuing, even though most vendors do not wear masks and PPE. This has led to an added risk of transmission in these communities. Those who walk to shops to place orders with water vendors are unable to practise physical distancing due to space constraints, intensifying the pathways of human-to-human transmission. Whilst everyone is urged to wash their hands with soap and water there is a lack of specific governmental WASH interventions targeting informal settlements. For that reason, local leaders have initiated and created additional handwashing facilities near shared areas such as communal washing areas, communal toilets, markets and places of worship.

Most of the households in Indonesian settlements use communal public toilets, as well as communal bathing and washing facilities. Given the space constraints and the number of people sharing the same spaces (in many places, over 20 families use the same facilities), it is challenging to practise physical distancing in those settings. Toilet guardians and cleaners usually do not wear masks or other PPE, leaving them highly vulnerable to infections. The situation in Indonesia is therefore somewhat similar to that of India, where public toilets pose a risk for added transmission of the disease.

In both countries, women carry the heavy burden of the lack of water and sanitation and risk added exposure to COVID-19 as a result of WASH-associated activities. Even though data indicates men suffer more virulent COVID-19 symptoms, to reduce transmission pathways for men it is essential to address the pathways which adversely affect women. This requires integrated gender-inclusive planning interventions tailored to all.

In addition to existing vulnerabilities created by the lack of access to adequate water and sanitation services, seasonal factors and climate change also have a role to play. With the lockdown occurring in the summer and an increased demand for water due to COVID-19, settlements in India will face water shortages during this time, limiting the ability to wash hands. Whilst water tankers are used to address this challenge in the short term, in the longer term measures to augment water resources and supply will be required. Purchasing water from tankers is only viable for those residents who can afford to pay for those services. COVID-19 requires increased water consumption for more frequent hand washing and improved hygiene practices placing an additional financial burden on households already struggling to pay for water services. Residents in our study settlements in India pay for water tankers whilst in Indonesia; the government reduced the price of clean water for communities during the pandemic to enable vulnerable communities to practise good hygiene during the pandemic. In both countries, the flooding of water streams and rivers in the rainy season will exacerbate solid and liquid waste contamination and result in a higher incidence of communicable diseases, which will further reduce the immunity of local populations and their ability to respond to and recover from COVID-19. There is therefore a need to combine COVID-19 mitigation measures with ongoing climate change mitigation and adaptation measures.

Moving forward, focused and dedicated health studies in informal settlements will be required to ensure the health and well-being of urban citizens, particularly in these settlements, as they are currently a hotbed of infections. Health studies of slums will need to include factors such as population densities, faecal contamination, water logging, crowding and hazards such as flooding and pollution [3]. Future monitoring and evaluation interventions will need to leverage existing social networks and use approaches such as citizen science combined with the testing of water sources and the testing of viral loading of wastewater. Working closely with existing social networks targeted public health campaigns will be required to empower communities to make informed and healthier choices.

The COVID-19 pandemic has made even clearer the need for improving the infrastructure of informal settlements and the implementation of contextualised public health interventions. In the short term, there is an urgent need for mobile hand-washing facilities with soap at regular points throughout these settlements, ideally per household or if not feasible at least at street level, and the added distribution of soap and portable communal washing/bathing facilities and toilets, possibly using local community centres and schools. Innovation is required to develop contactless solutions for hand-washing stations such as sensors, foot pedals and long handles to turn taps on and off. Contactless soap dispensers and safe places to store soap would further help to ensure the usage of soap [10]. There is scope to learn lessons from the humanitarian settings where low-cost, low-tech and portable hand-washing kits have been developed by organisations such as Oxfam [11]. Additionally, water vendors, shopkeepers and sanitation workers need to be provided with PPE for both their safety and the safety of others.

In the long term, radical changes are required in city planning, as informal settlements are currently bypassed by mainstream infrastructure interventions. Even in the pre-pandemic era slum residents perceived water and sanitation to be their top priority [12]. Hence, moving forward, targeted investment is required to improve access to water and sanitation services in these settlements, as well as river/blue space cleaning and restoration activities. In fact, the high population density of such areas offers economies of scale, high returns to infrastructure investments and creates healthy environments [13].

COVID-19 provides an opportunity to invest in centralised water and sanitation-networked solutions appropriate for high-density settings. This would facilitate the integration of those settlements into mainstream cities, and improve environmental conditions and health in these cities. As informal settlements provide the work force of cities – higher risks of transmission of infections in those communities also poses a risk for other parts of the city. That being said, targeted environmental improvements will increase the ability of cities to respond to pandemics and the health of local populations with a reduction in transmission pathways to infections.

## Data Availability

Data sharing not applicable to this article as no datasets were generated or analysed during the current study.
